# Expanding the Analytical Toolbox: Developing New Lys-C Peptide Mapping Methods with Minimized Assay-Induced Artifacts to Fully Characterize Antibodies

**DOI:** 10.3390/ph16091327

**Published:** 2023-09-20

**Authors:** Y. Diana Liu, Michelle Irwin Beardsley, Feng Yang

**Affiliations:** Department of Protein Analytical Chemistry, Genentech/Roche, South San Francisco, CA 94080, USA; dliu1@gene.com (Y.D.L.); michelli@gene.com (M.I.B.)

**Keywords:** biopharmaceuticals, drug substances, sample preparation, enzymatic digestion, mass spectrometry

## Abstract

Peptide mapping is an important tool used to confirm that the correct sequence has been expressed for a protein and to evaluate protein post-translational modifications (PTMs) that may arise during the production, processing, or storage of protein drugs. Our new orally administered drug (Ab-1), a single-domain antibody, is highly stable and resistant to proteolysis. Analysis via the commonly used tryptic mapping method did not generate sufficient sequence coverage. Alternative methods were needed to study the Ab-1 drug substance (75 mg/mL) and drug product (3 mg/mL). To meet these analytical needs, we developed two new peptide mapping methods using lysyl endopeptidase (Lys-C) digestion. These newly developed protein digestion protocols do not require desalting/buffer-exchange steps, thereby reducing sample preparation time and improving method robustness. Additionally, the protein digestion is performed under neutral pH with methionine acting as a scavenger to minimize artifacts, such as deamidation and oxidation, which are induced during sample preparation. Further, the method for low-concentration samples performs comparably to the method for high-concentration samples. Both methods provide 100% sequence coverage for Ab-1, and, therefore, enable comprehensive characterization for its product quality attribute (PQA) assessment. Both methods can be used to study other antibody formats.

## 1. Introduction

Biotherapeutic drugs, especially antibodies, dominate pharmaceutical pipelines due to their suitability for modulating extracellular targets with high potency and selectivity [1,2,3]. Recent advances in drug discovery have allowed us to witness the significant growth of next-generation antibodies [4,5,6]. These new antibody formats, which include bispecific (multispecific) antibodies, nanobodies, antibody fragments, and modified antibodies, could help to address unmet medical needs and provide treatments for cancer and inflammatory or immunological diseases [1,3]. Understanding the structural aspects of these new antibody formats is essential, because certain structural characteristics are strongly associated with the safety and efficacy of the antibodies, thus affecting the drugs’ critical qualities [7,8,9,10,11,12,13,14,15,16]. Despite the existence of a variety of analytical methods historically used to characterize biotherapeutics [17,18,19,20,21], next-generation antibodies require innovative analytical methods to characterize and monitor drug quality attributes, as new drug formats present new types of analytical challenges.

At the core of many analytical strategies, peptide mapping methods have been widely used for the structural characterization of antibody-based therapeutics. Few other assays can claim to match peptide mapping for sensitivity and specificity [2]. A typical peptide mapping workflow includes the enzymatic digestion of a protein into peptides, peptide mixture separation and detection via liquid chromatography (LC) coupled with high-resolution accurate mass (HRAM) mass spectrometry (MS), and data analysis using cutting-edge software tools. This analysis can provide specific and structurally resolved information on protein sequence, post-translational modifications (PTMs), and process impurities, all of which are important in molecule critical quality attribute (CQA) assessment, cell line selection, production process optimization, stability and release testing, and extended characterization [22]. Peptide mapping combined with new-peak detection (NPD) allows for the simultaneous monitoring of multiple product quality attributes (PQAs) in a single analysis. This powerful approach is known as the multi-attribute method (MAM). NPD enables new impurities (displayed as new peaks) to be detected when compared with a reference sample [23]. MAM has the potential to replace several conventional methods, thus increasing the efficiency of analytical testing throughout all stages of these biopharmaceutical drug development processes [23,24,25].

One of the key components of MAM is a well-optimized and reproducible protein digestion. The digestion is performed with specific proteases. The most commonly used protease for conventional antibodies is trypsin, because it produces peptides in the size range that is most efficient for peptide identification via MS analysis [23,26]. Our MAM platform method uses trypsin as a digestion enzyme [27]. However, alternative protein digestion using different enzymes might be more desirable for specific applications, in order to obtain complete sequence coverage [28,29,30]. For example, trypsin digestion is not suitable for the new pipeline molecule, Ab-1, which targets inflammatory bowel disease. Ab-1 is highly stable and tolerates extreme temperature and pH conditions, which makes it a good candidate for oral administration via tablet or capsule. Most importantly, based on the amino acid sequence of Ab-1, tryptic digestion (in silico) generates three small peptides near its complementarity-determining regions (CDRs) that will not be captured in the analysis, resulting in sequence coverage of 91%. An alternative enzyme digestion protocol must be developed using a different enzyme and procedures to accommodate these analytical needs. Lysyl endopeptidase (Lys-C), which retains proteolytic activity and specificity under strong protein-denaturing conditions [28], can be used to improve the digestion of proteolytic-resistant proteins. Based on the in silico analysis of the Ab-1 amino acid sequence using Lys-C, complete sequence coverage is possible for this challenging molecule. A recently improved MAM with reduced Lys-C digestion was reported for the analysis of therapeutic monoclonal antibodies (mAbs) [31], in which the Lys-C digestion protocol eliminated the desalting step and a specific column was used for enhanced product quality attribute monitoring of hydrophilic peptides; its analytical performance was evaluated [32]. However, the digestion was carried out at pH 8.0, which is known to introduce sample-preparation-related artifacts (e.g., increased deamidation). Other artifacts such as oxidation might be introduced as well. Moreover, the reported method is not suitable for low-concentration protein drugs. Therefore, a robust digestion method is needed to characterize protein drugs at high and low concentrations with minimized method-introduced artifacts in PQA characterization.

In this work, we developed optimized Lys-C digestion protocols using the amino acid methionine (Met) as a scavenger and a neutral pH to minimize the artifacts induced during protein digestion. Additionally, we were able to establish a method for low-concentration proteins with comparable performance to those of high-concentration protocols. Two new Lys-C digestion protocols were established to accommodate different concentrations of the Ab-1 drug substance (DS; 75 mg/mL) and drug product (DP; 3 mg/mL). These Lys-C peptide mapping methods enable comprehensive characterization and monitoring of PQAs. They also serve as alternative or complementary methods to the MAM platform method (tryptic mapping) used for many antibodies. Here, we also highlight the impact of our newly developed characterization methods and their applications in drug stability studies. These new methods are valuable additions to our analytical toolbox for antibody characterization beyond traditional modalities. Compared to previous methods, our newly developed methods not only minimize method-introduced artifacts in PQA characterization, but also provide robust digestion for both high- and low-concentration protein drugs.

## 2. Results and Discussion

### 2.1. Method Development: Lys-C Digestion Method 1 

Lys-C digestion Method 1 was developed to be suitable for Ab-1 DS or any protein sample with a concentration ≥ 50 mg/mL. Our experimental design covered several considerations for the enzymatic digestion of the proteolysis-resistant protein Ab-1. (1) In this method, Ab-1 was denatured in 6 M guanidine HCl (GuHCl), reduced with dithiothreitol (DTT), and carbamidomethylated using iodoacetamide (IAM). (2) We chose DTT over tris(2-carboxyethyl)phosphine (TCEP) as the reducing agent because TCEP can reduce oxidized Met residues [33] on the molecule prior to digestion, resulting in less accurate quantitation of that attribute. (3) The most commonly used alkylation reagents are iodoacetic acid (IAA) and IAM. We used IAM because IAA is typically dissolved in 1 N NaOH, which would increase the pH of the sample mixture and introduce a negative charge to peptides [34,35]. (4) The reduced and alkylated protein was digested with Lys-C enzyme after a simple dilution step to lower GuHCl to ≤2 M. GuHCl concentrations greater than 2 M affect enzyme activity. At a concentration ≤ 2 M GuHCl, Lys-C retains 90% of its activity while keeping the protein in a more denatured condition to help digestion of the protease-resistant Ab-1, whereas trypsin retains only 50% of its activity and requires a desalting/buffer-exchange step to remove GuHCl prior to protein digestion [36,37]. Therefore, with a dilution step rather than a desalting/buffer-exchange, this Lys-C digestion protocol was simplified without the need for the additional desalting step. 

To optimize the Lys-C digestion conditions, we focused on the following parameters: reagent concentration, buffer pH, incubation time and temperature during reduction, alkylation, and digestion. Table 1 provides a detailed list of the conditions tested. It is important that the antibody is sufficiently denatured, unfolded, and reduced prior to digestion, allowing Lys-C to easily access the molecule for a complete digestion. To ensure that the protein’s reduction would break its disulfide linkages and improve its digestion, we optimized the level of the reducing agent DTT to achieve complete reduction without an excess of DTT. We examined the amount of time required to fully reduce the protein, and we evaluated and selected a pH that would ensure complete reduction and minimize artificial deamidation. The optimal condition determined for each parameter is shown as a bold, underlined value in Table 1. The best reduction conditions were achieved using 3.5 mM DTT and pH 7.5 at 37 °C for 1 h. Following reduction, alkylation of the sulfhydryl groups on the cysteine residues was required to prevent reformation of the disulfide bonds. IAM was used in a 2.5-fold excess of DTT to ensure complete alkylation. However, if too much IAM was used, it would overalkylate the protein, which would result in the alkylation of amino acid residues other than cysteine (e.g., lysine). We tested the alkylation conditions, including IAM concentration and incubation time. The optimized conditions for alkylation were 8.5 mM IAM for 15 min at 37 °C in the dark. The remaining (minimal) IAM after reaction with DTT was unstable under light and in the presence of excess water from a 3-fold dilution immediately after the alkylation step. Therefore, there was no need to use additional DTT to quench the alkylation step.

As this protocol eliminated the desalting step, a simple dilution of the sample into the digestion buffer lowered the GuHCl concentration (≤2 M). Two dilution schemes were examined, as shown in Table 1. Between them, Dilution Scheme 1, with a 3-fold dilution (before digestion), performed well, while the digestion efficiency was decreased when a 9-fold dilution was used (Dilution Scheme 2). After the dilution, the Lys-C digestion was carried out at pH 7 to minimize artificial deamidation occurring during protein digestion [38]. Lys-C enzyme to protein ratios (enzyme: protein *w*/*w*) of 1:20 and 1:10 and digestion times were evaluated. The results revealed that efficient protein digestion was achieved after 2 h of Lys-C digestion at 37 °C. One important consideration when developing such a method is to choose the conditions that will minimize the degree of artificial modifications induced, balancing maximum digestion efficiency with minimum artificial modifications. Besides digestion at neutral pH for minimal artificial deamidation, free Met was added as a scavenger throughout the sample preparation to reduce artificial oxidation [39]. 

### 2.2. Method Development: Lys-C Digestion Method 2

The Ab-1 DP is formulated in a capsule form, i.e., an enterically coated solid form. After dissolving the capsule in liquid (90% 20 mM sodium phosphate buffer pH 7: 10% methanol, *v*:*v*) for analysis, the concentration was 3 mg/mL. With the protein at such a low concentration, any digestion using a dilution scheme was especially challenging, as the protein concentration in the digestion step was very low (0.03 mg/mL) if Method 1 was used. Our main focus was to find lower concentrations of the denaturing agent that were still effective for protein denaturing, so that a smaller dilution factor could be used. Therefore, lower concentrations of GuHCl (2–5 M) were tested, and the minimum concentration used to get complete reduction was 4.7 M. Another method modification was to dissolve DTT in Tris HCl buffer instead of water to avoid further dilution of the sample. Other conditions remained the same as those used in Method 1, including reduction and alkylation reagent concentrations, Lys-C enzyme-to-protein ratio, and incubation times. In parallel, Table 2 lists the reagent and protein concentrations after each step for both methods. Using Method 2, the final protein concentration after the digestion was 0.2 mg/mL. Note that the Met levels were maintained in the range of 10–20 mM to prevent protein oxidation during the sample preparation [39]. As the recommended injection amount for a protein sample is 2.5–5.0 µg for LC/MS analysis, the injection volume was modified accordingly. This Lys-C digestion protocol was suitable for use with Ab-1 DP lots or for samples with low concentrations (≥3 mg/mL).

### 2.3. Method Performance Evaluation

To evaluate the peptide mapping method’s performance, we focused on several aspects, namely protein reduction completion, alkylation efficiency, and digestion completion. Disulfide bonds are present in many proteins, including Ab-1. Protein reduction and alkylation conditions play important roles in the complete reduction of disulfides. When optimizing the conditions for protein reduction, the reduction completion of disulfides was assessed and confirmed. Alkylation efficiency was also thoroughly evaluated. Efficiency decreased when the alkylation of cysteine was not complete, i.e., <100% cysteine was alkylated. Overalkylation was measured when the N-terminus or other amino acids besides cysteine (e.g., lysine) were alkylated. Therefore, we adjusted the alkylating agent and incubation time to balance under- and overalkylation. The average alkylation efficiency was measured to be >95%. Optimizing reduction and alkylation conditions was also essential for improving protein digestion. 

**Figure 1:**
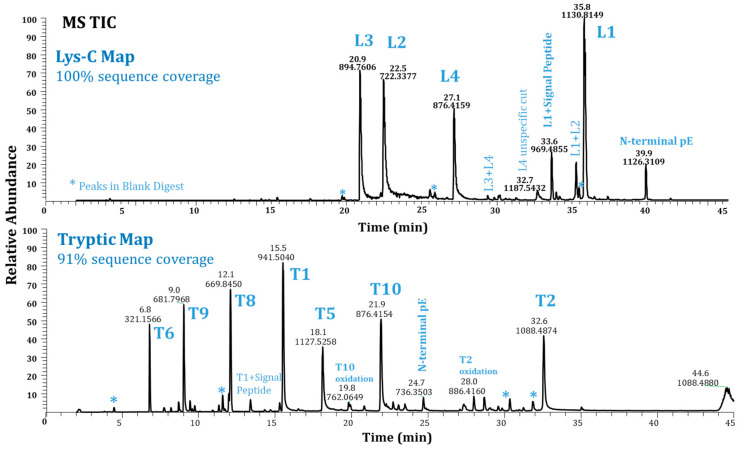
MS total ion chromatograms of Ab-1 via Method 1 (L1–L4 = peptides by Lys-C, T1–T10 = peptides by trypsin, missing T3, T4, and T7).

Protein sequence coverage is the key measurement when evaluating the performance of a peptide mapping method. In this study, the digestion conditions were optimized to achieve maximized protein sequence coverage. In Figure 1, the top panel is the MS total ion chromatogram (TIC) of Lys-C-digested Ab-1, which generated four peptides (L1–L4) covering 100% of the sequence. In comparison, the bottom panel shows the TIC of the Ab-1 trypsin digest; only 7 of 10 tryptic peptides (T1–10) were observed, resulting in 91% sequence coverage. The three missed peptides (T3, T4, and T7) are peptides of only a few residues (five, two, and four amino acids) and are relatively more hydrophilic; they thus were elute at the void volume and are not detected. Therefore, Lys-C was the enzyme choice for Ab-1, as it generated the 100% sequence coverage that was expected. 

However, the biggest challenge associated with the use of Lys-C digestion for this molecule was the high level of missed cleavage peptides due to the enzymatic resistance of the molecule. A missed cleavage occurred at the lysine residue of Peptide L1, leading to a bigger peptide, L1 + L2, which ranged from 9% to 15% in different sample preparations by different analysts or on different days. Such a high level of missed cleavage caused an inconsistency in method quantitation. Note that other peptides with a missed cleavage (L3 + L4 and L2 + L3) were below 2%, which was less of a concern. Figure 1’s top panel shows TIC chromatograms analyzed using the newly developed Lys-C peptide mapping method (Method 1) and illustrates the protein’s wild-type peptides L1 to L4 with some miscleaved peptide peaks. Note that peptides with missed cleavages are common in peptide mapping. They are useful in some cases to improve sequence coverage. For example, a short peptide, usually three amino acids and less, will be too hydrophilic to be retained in a reversed-phase (RP) high-performance liquid chromatography (HPLC) column. It will often be washed out in the void volume with the small-molecule reagents used in sample preparation. With a peptide missed cleavage event, these short peptides can be retained in an RP column and observed in LC/MS analysis when they attach to other peptides. Therefore, the protein sequence coverage is improved. In contrast to miscleavages from incomplete digestion, overdigestion of a protein produces unspecific enzyme cuts, which are also undesirable. In Figure 1, the peak with *m*/*z* 1187.5432 is identified as Peptide L4 with an enzyme cut at a nonlysine residue, and is labeled as “L4 unspecific cut”. To minimize both miscleavages and unspecific cuts, we carefully optimized the digestion conditions and carried out tests with higher amounts of enzyme and longer digestion times. In the end, with the optimized digestion conditions, we were able to bring down the level of the miscleaved peptide L1 + L2 to less than 5% from the 9–15% range.

**Figure 2:**
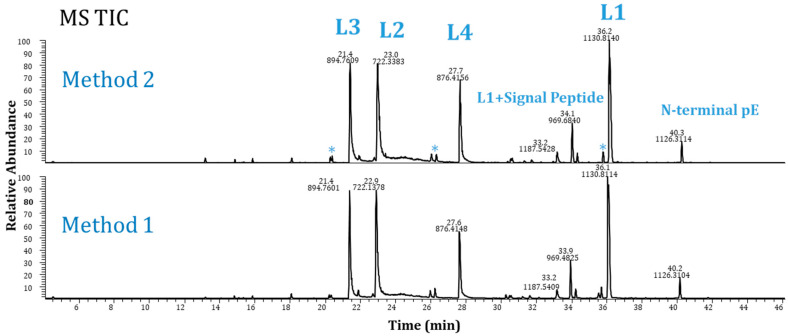
MS total ion current chromatograms of Ab-1: Method 1 (bottom) and Method 2 (top). * Peaks in blank digest. (L1–L4 = peptides from Lys-C digestion).

**Figure 3:**
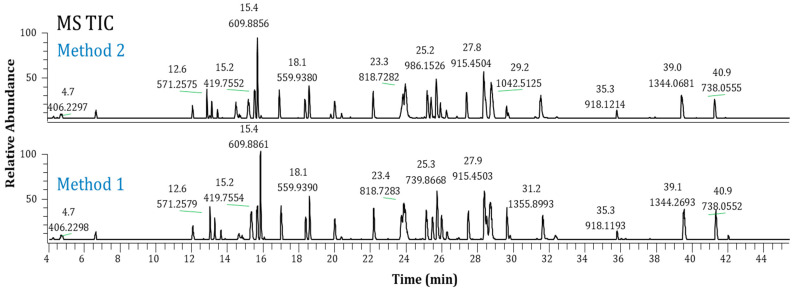
MS total ion current chromatograms of Ab-2: Method 1 (bottom) and Method 2 (top).

Figure 2 displays typical MS TIC chromatograms of Lys-C-digested Ab-1 following the Method 1 and Method 2 protocols. Both methods provided comparable results, providing a choice for analysts to use. Moreover, using these methods, we tested seven other molecules, including mAbs, bispecific antibodies, an antibody drug conjugate, and a Fab antibody fragment. Figure 3 shows the TIC chromatograms of Ab-2 (a mAb) as an example. The sequence coverages were 100% for Ab-1 and 96% for Ab-2. In addition, six other antibodies were tested with good protein sequence coverages (93–98%). These results demonstrate the broader utility of these methods. However, the choice of enzyme for digesting a particular molecule should be considered carefully based on the specific situation and sequence of each molecule. Therefore, these two newly developed Lys-C mapping methods can be applied to various antibody formats.

### 2.4. Method Robustness

The optimized Lys-C digestion methods were evaluated and demonstrated to be robust. To assess the robustness, a half fractional factorial design of experiment (DoE) with five factors and two levels was performed (Table 3). Digest buffer pH, time, temperature, and reduction buffer pH were included because they are known to affect deamidation, pyroglutamate (pE) formation, isomerization, and succinimide formation. Injection volume changes affect quantitation as sample load changes; therefore, this was also included in the DoE study. A total of 20 runs, including an additional 2 replicates and 2 center points, were completed for each method.

The DoE study demonstrated the robustness of Method 1 and Method 2. Table 4 lists the statistics for the DoE study with relative abundance maximum (Max), minimum (Min), difference (Max-Min), average, standard deviation (SD), and relative SD (%RSD). Based on these results, both methods are robust for Ab-1 PTM quantification, as the RSD was 5% or less.

### 2.5. Suitability for Ab-1 Stability Study

**Figure 4:**
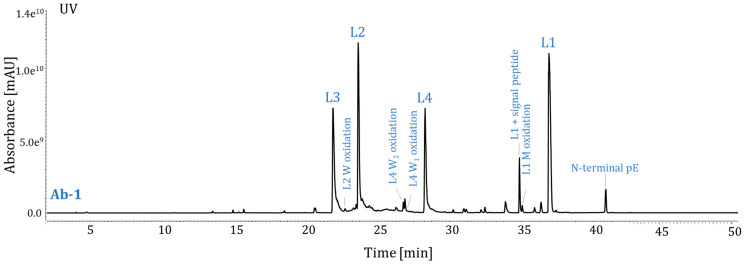
UV chromatograms of the protein digest of Ab-1 (L1–L4 = peptides from Lys-C digestion; prepared using Method 1). M, methionine; W, tryptophan.

The major PTMs of Ab-1 from the manufacturing process were the N-terminal signal peptide and N-terminal pE. In the stability studies, Ab-1 samples were subjected to chemical and physical stresses including oxidation by 2,2-azobis (2-amidinopropane) dihydrochloride (AAPH), elevated temperatures, and low pH. AAPH is a free radical generator that produces alkoxyl and alkyl peroxyl radicals. The AAPH stress model is used as an indicator of oxidation susceptibility for antibodies. Such stress often induces protein oxidation of Met or tryptophan residues [8,9]. Stress under elevated temperatures and low pH may produce protein deamidation of asparagine [7,17], pE formation at N-terminal glutamic acid [19,20], etc. Even if it is not known whether these PTMs could affect biological activity, monitoring PTMs occurs as part of routine quality testing because such quality control testing can ensure the safety and efficacy of DPs. Using the newly developed Lys-C maps, we identified and quantified these modifications of Ab-1. Figure 4 illustrates the protein’s wild-type peptides L1–L4 with well-separated PTM-modified peptides. 

The Lys-C digestion method is stability-indicating. Unstressed (control) and stressed samples were analyzed in parallel using the new method. The results of the force-stress testing of Ab-1 DS samples using AAPH, high temperatures, and low pH are summarized in Table 5. The Ab-1 samples subjected to thermal stress conditions (storage at 40 °C and 50 °C) demonstrated an increase in N-terminal pE compared to the control sample. Deamidation was not detected at either of the two asparagine residues of Ab-1. It is worth noting that Ab-1 is particularly tolerant to high temperatures, and, therefore, significant degradation upon thermal stress was not expected. The Ab-1 samples subjected to 10 mM AAPH demonstrated an increase in oxidation at two tryptophan residues (W_1_, W_2_) in peptide L4 compared to the unstressed sample. No significant increase in oxidation was observed at other tryptophan or Met residues. The analysis of stressed samples demonstrated that the Lys-C digestion method is stability-indicating for attributes at critical sites for Ab-1. Relative levels of induced modifications were calculated as follows:

$$\%\;\mathrm{Attribute}=\frac{\sum \;\mathrm{Peak}\;\mathrm{Areas}\;\mathrm{of}\;\mathrm{modified}\;\mathrm{peptides}}{\sum \;\mathrm{Peak}\;\mathrm{Areas}\;\mathrm{of}\;\mathrm{wild}\;\mathrm{type}\;\&\;\mathrm{modified}\;\mathrm{peptides}}\times \;100$$

## 3. Materials and Methods

### 3.1. Materials

This study used a camelid single-variable domain on a heavy-chain antibody (a fragment nanobody), Ab-1, which is a variable domain (VH) of a heavy-chain (HC) antibody. Ab-1 is derived from llama immunoglobulin G (IgG) and purified using the fed batch platform cell culture procedure without an affinity step. Ab-2 is a mAb derived from Chinese hamster ovary (CHO) cells. Both antibodies were made at the Roche/Genentech facilities. Pierce™ dithiothreitol (DTT), Pierce iodoacetamide (IAM), Pierce formic acid (FA), Pierce trifluoroacetic acid (TFA), and 8 M guanidine HCl (GuHCl) were obtained from Thermo Fisher Scientific (Sunnyvale, CA, USA). Lysyl endopeptidase^®^ MS grade (Lys-C) was purchased from Wako Chemicals (Richmond, VA, USA). L-methionine (Met) and AAPH (2,2’-azobis (2-amidinopropane) dihydrochloride) were from Sigma-Aldrich (St. Louis, MO, USA). All organic solvents were of analytical or HPLC grade.

### 3.2. Protein Digestion

#### 3.2.1. Method 1: Lys-C Digestion for High-Concentration Samples (≥50 mg/mL)

The optimized Lys-C digestion protocol for 50 mg/mL samples was as follows: in a sample tube, 50 mg/mL protein (12 µL) was mixed with 85 µL of 8 M GuHCl and 6 µL of 0.2 M Met in 1 M Tris HCl (pH 7.5). The reduction was started by adding 10 µL of 40 mM DTT. After 1 h incubation at 37 °C, alkylation was carried out by adding 5 µL of 200 mM IAM and incubating at 37 °C in the dark for 15 min. Then, an aliquot of 25 µL of the reduced/alkylated protein (125 µg) was removed to a new tube, in which the sample was diluted by adding 39 µL of water and 6 µL of 0.2 M Met in 1 M Tris HCl (pH 7.0). To digest the protein, 13 µL of 0.5 mg/mL Lys-C solution was added to the sample mixture (enzyme-to-substrate weight ratio of 1:20), and the sample was incubated at 37 °C for 2 h. After digestion, the sample was further diluted with 140 µL of water and 20 µL of 0.2 M Met, and the protein digestion was quenched with the addition of 5 µL of 10%TFA. The digested protein mixture was frozen until analysis. For LC/MS analysis, the injection volume was 5 µL (2.5 µg Ab-1).

#### 3.2.2. Method 2: Lys-C Digestion for Low-Concentration Samples (≥3 mg/mL)

In a sample tube, 3 mg/mL protein (40 µL) was mixed with 67 µL of 8 M GuHCl and 6 µL of 65 mM DTT, 0.2 M Met in 1 M Tris HCl (pH 7.5). Protein reduction was carried out at 37 °C with a 1 h incubation. Then, alkylation was carried out by adding 5 µL of 200 mM IAM and incubating at 37 °C in the dark for 15 min. A 68 µL aliquot of the reduced/alkylated protein (68 µg) was removed to a new tube, in which the sample was diluted by adding 74 µL of water and 12 µL of 0.2 M Met in 1 M Tris HCl (pH 7.0). To digest the protein, 7 µL of 0.5 mg/mL Lys-C solution was added to the sample mixture (enzyme-to-substrate weight ratio of 1:20) and the sample was incubated at 37 °C for 2 h. After digestion, 79 µL of the digested sample was pipetted into a new sample tube and diluted with 120 µL of water and 20 µL of 0.2 M Met. The protein digestion was quenched with the addition of 5 µL of 10% TFA. The digested protein mixture was frozen until analysis. For LC/MS analysis, the injection volume was 17 µL (2.5 µg Ab-1). 

### 3.3. LC/MS Analysis

LC/MS analysis was performed using a Q Exactive™ Hybrid Quadrupole-Orbitrap™ mass spectrometer coupled with a Vanquish ultra-high-performance liquid chromatography (UHPLC) system (Thermo Fisher Scientific, Sunnyvale, CA, USA). A separation column, Acquity Premier Peptide CSH C18 Column 1.7 µm, 2.1 × 150 mm (Waters Corporation, Milford, MA, USA), was used with a column temperature of 77 °C. The mobile phases A and B consisted of 0.1% FA in water and 0.1% FA in acetonitrile (ACN), respectively. The flow rate was set to 0.2 mL/min. The pump gradient was as follows: an isocratic flow at 1% B for 8 min, and a multistep linear gradient from 1% B to 13% B for 5 min, 13% B to 35% B for 35 min, and 35% B to 95% B for 2 min. The column wash and equilibration was a step-wash, repeated five times, with an isocratic flow at 95% B for 2 min and a sharp gradient from 95% B to 1% B in 2 min. The total run time was 86 min. The MS scan type was full MS with data-dependent MS^2^ (dd-MS^2^) for the top eight highest intensity ions. The electrospray voltage was 3.5 kV in positive ion mode. Both capillary and aux gas heater temperatures were at 250 °C. The full mass scan range was 200–2000 (*m*/*z*) with full mass resolution at 35k and dd-MS^2^ resolution at 17.5 k. The collision energy for data-dependent acquisition was high-energy collisional dissociation. The MS data collection started at 4 min and ended at 45 min for a total 86 min run time. The acquisition was controlled by the vendor’s Chromeleon™ software (Thermo Fisher Scientific, Sunnyvale, CA, USA).

### 3.4. MS Data Analysis

The raw MS files were analyzed using the Byos^®^ software suite (Protein Metrics Inc., Cupertino, CA, USA). Peptides were identified via searching against the Ab-1 sequence using the accurate mass of a full mass scan and assignments of product ions in MS^2^ spectra. The cutoff MS^2^ score was 250 and the maximum precursor m/z error was ±10ppm. Carbamidomethylation (+57.0215 Da) was set as a fixed modification of cysteine residues. The modification list included oxidation (+15.9949 Da) of Met and tryptophan, deamidations (+0.9840 Da) of asparagine, pE (−18.0106 Da) of N-terminal glutamic acid, signal peptide at protein N-terminus (+323.1594), glycation (+162.0528) of lysine, carbamidomethylation (+57.0215 Da) of lysine, and de-carbamidomethylation (−57.0215 Da) of cysteine. The modification percent per peptide in the software’s PTM default report was used to report the detected levels of PTMs. 

### 3.5. Tryptic Peptide Map

The trypsin digest was performed following the protocols described in a previous publication [40]. LC/MS analysis was conducted using the same procedure as described in Section 3.3.

## 4. Conclusions

To support the characterization of a new pipeline molecule, Ab-1, we developed two new Lys-C peptide mapping methods. Method 1 is suitable for relatively higher-concentration protein samples such as Ab-1 DS (≥50 mg/mL), and Method 2 for low-concentration (≥3 mg/mL) samples such as Ab-1 DP. These methods were optimized using a neutral pH digestion buffer (pH 7) and a short digestion time (2 h) to minimize artificial deamidation and pE formation. Free Met was added throughout the sample preparation as a scavenger to reduce artificial oxidation. The protein digestion protocols without the desalting steps increased the sample preparation throughput and minimized process-induced modifications. Using these simple and reliable protein digestion procedures, we were able to analyze Ab-1 with 100% sequence coverage and good PTM peptide separations. Furthermore, we demonstrated the robustness of the new methods via well-designed DoE studies. These methods were also successfully applied to a protein stability study, showing differences at the peptide level between stressed and unstressed materials. As a well-optimized Lys-C digestion is one of the key components of MAM, these new methods lay the foundation for future applications in building Lys-C MAM methods. We also applied these new methods to various antibody formats. Therefore, the methods are valuable additions to our analytical toolbox for antibody characterization.

## Figures and Tables

**Table 1:** Method 1: optimization of test conditions.

	Parameter	Test Condition ^a^	Note
Reduction	DTT (mM)	**3.5**, 5	6 M GuHCl for protein denaturation at 37 °C. Use Met (10–20 mM) as a scavenger throughout the procedure to reduce artificial oxidation
	Buffer pH	7, **7.5**, 8	
	Incubation time (min)	30, 45, **60**, 90	
Alkylation	IAM (mM)	6.8, **8.5**	Alkylation at 37 °C in the dark
	Incubation time (min)	**15**, 30	
Dilution	Scheme 1. Dilute 3× before digestion and then dilute 3× before LC/MS injection Scheme 2. Dilute 9× before digestion		Dilution to lower GuHCl level (≤2 M) in digestion and more dilution (<1 M) before LC/MS injection
Digestion	Lys-C enzyme-to-protein ratio	**1:20**, 1:10	Digestion at pH 7 to minimize artificial deamidation Digestion temperature: 37 °C
	Incubation time	30 min, 1 h, **2 h**	

^a^ Bold, underlined value indicates the optimal test condition. DTT, dithiothreitol; GuHCl, guanidine HCl; IAM, iodoacetamide; LC/MS, liquid chromatography–mass spectrometry; Met, methionine.

**Table 2:** Stepwise procedures and the concentrations of the protein sample and reagents.

	Method 1	Reagent	Concentration	Method 2	Reagent	Concentration
Reduction	8 M GuHCl 85 µL	GuHCl	6.0 M	8 M GuHCl 67 µL	GuHCl	4.7 M
	1 M Tris pH 7.5/0.2 M Met 6 µL	Tris HCl	0.05 M	1 M Tris HCl pH 7.5/0.2 M Met/65 mM DTT 6 µL	Tris HCl	0.05 M
		Met	11 mM		Met	11 mM
	40 mM DTT 10 µL	DTT	3.5 mM		DTT	3.5 mM
	50 mg/mL Ab–1 12 µL	protein	5.1 mg/mL	3 mg/mL Ab–1 40 µL	protein	1.0 mg/mL
	Incubate at 37 °C for 1 h			Incubate at 37 °C for 1 h		
Alkylation	200 mM IAM 5 µL	IAM	8.5 mM	200 mM IAM 5 µL	IAM	8.5 mM
	Incubate at 37 °C for 15 min in the dark			Incubate at 37 °C for 15 min in the dark		
Digestion	Take 25 µLof the alkylated sample (125 µg) into a new tube			Take 68 µL of the reduced sample (68 µg) into a new tube		
	Reduced sample 25 µL	protein	1.5 mg/mL	Reduced sample 68 µL	protein	0.4 mg/mL
	Add H_2_O 39 µL (GuHCl conc. < 2 M)	GuHCl	1.8 M	Add H_2_O 74 µL (GuHCl conc. < 2 M)	GuHCl	2.0 M
	1 M Tris pH 7/0.2 M Met 6 µL	Tris HCl	0.1 M	1 M Tris HCl pH 7/0.2 M Met 12 µL	Tris HCl	0.1 M
		Met	18 mM		Met	19 mM
	0.5 mg/mL Lys-C solution 13 µL	Lys-C: protein	1:20 (*w*:*w*)	0.5 mg/mL Lys-C solution 7 µL	Lys-C: protein	1:20 (*w*:*w*)
	Incubate at 37 °C for 2 h			Incubate at 37 °C for 2 h		
Dilution & Quench	Dilution (3×)	protein	0.5 mg/mL	Pipette 79 µL digested sample, dilute 3×	protein	0.2 mg/mL
	Add H_2_O 140 µL (GuHCl conc. < 1 M)	GuHCl	0.6 M	Add H_2_O 120 µL (GuHCl conc. < 1 M)	GuHCl	0.7 M
	Add 20 µL 0.2 M Met	Met	16 mM	Add20 µL 0.2 M Met	Met	18 mM
	Add 10% TFA 5 µL to quench digestion	TFA	0.2%	Add 10%TFA 5 µL to quench digestion	TFA	0.2%

Notes: Method 1 was developed for protein samples with concentration of 50 mg/mL. For protein samples with higher concentrations (e.g., Ab-1 DS, 75 mg/mL), the samples were diluted to 50 mg/mL with their respective formulation buffers before protein digestion. In the table: DTT, dithiothreitol; GuHCl, guanidine HCl; IAM, iodoacetamide; Met, methionine; TFA, trifluoroacetic acid.

**Table 3:** The DoE design with factors and levels.

DoE Factor	Condition Specifications	DoE Level (Low/High)
Digest buffer (pH) ^a^	7.0 ± 0.1	6.9/7.1
Digest time (min) ^a^	120 ± 10	110/130
Digest temperature (°C) ^a^	37 ± 2	35/39
Reduction buffer (pH) ^a^	7.5 ± 0.1	7.4/7.6
Injection volume (µL) ^b^	5 for Method 1	4/6
	17 for Method 2	15/19

^a^ This DoE factor can affect deamidation, pE formation, isomerization, and succinimide. ^b^ This DoE factor can affect quantitation as the load increases.

**Table 4:** DoE results: the statistics of relative abundance for Method 1 and Method 2.

Statistics	Method 1		Method 2	
	Signal Peptide (%)	N-Terminal pE (%)	Signal Peptide (%)	N-Terminal pE (%)
Max	13.2	7.1	13.9	7.3
Min	11.7	6.3	11.9	6.1
Difference (Max-Min)	1.5	0.8	2.0	1.2
Average	12.5	6.7	12.8	6.7
SD	0.4	0.2	0.5	0.3
% RSD	3.3%	2.8%	4.2%	5.1%

Min, minimum; Max, maximum; pE, pyroglutamate; SD, standard deviation; %RSD, relative SD.

**Table 5:** Attribute quantitation of the stressed Ab-1 DS (via Method 1).

Attribute	Relative Abundance (%)						
	t = 0	4 Weeks at 40 °C	2 Weeks at 50 °C	pH Control	pH 3.25	AAPH Control	10 mM AAPH
L1 + Signal peptide	9.6	9.0	9.3	10.3	10.2	10.6	10.0
N-terminal pE	5.7	7.3	9.5	6.4	7.0	7.3	6.7
L1 M oxidation	2.3	2.3	2.2	2.2	2.1	2.2	2.1
L1 W oxidation	<0.1	<0.1	<0.1	<0.1	<0.1	<0.1	<0.1
L2 W oxidation	<0.1	ND	ND	0.1	0.2	0.1	1.1
L3 N_1_ deamidation	ND	ND	ND	ND	ND	ND	ND
L3 N_2_ deamidation	ND	ND	ND	ND	ND	ND	ND
L4 W_1_ oxidation	0.3	0.3	0.4	0.3	0.2	0.3	3.7
L4 W_2_ oxidation	1.3	1.0	1.3	0.9	0.8	0.9	11.2

AAPH, 2,2’-azobis (2-amidinopropane) dihydrochloride; L1–L4 = peptides from Lys-C digestion; ND, not detected; W, tryptophan.

## Data Availability

Data is contained within the article.
